# The impact of small groups on pre‐ and postcopulatory sexual selection in polyandrous populations

**DOI:** 10.1002/ece3.10057

**Published:** 2023-05-02

**Authors:** Grant C. McDonald

**Affiliations:** ^1^ Department of Ecology University of Veterinary Medicine Budapest Budapest Hungary

**Keywords:** polyandry, sexual network, sexual selection, social organization, sperm competition

## Abstract

Sexual selection is a key evolutionary force but varies widely between populations. Two key factors that influence sexual selection are the extent to which females copulate with multiple males (polyandry) and variation in the social environment. Increasing research demonstrates populations are structured by complex socio‐sexual networks, and the structure of these networks can influence sexual selection by shaping the relationship between male precopulatory mating success and the intensity of postcopulatory competition. However, comparatively less attention has been dedicated to the influence of group structure on sexual selection and how differences in the size of groups may impact on the relative force of pre‐ and postcopulatory sexual selection in polyandrous populations. The presence of groups (i.e., group structure) and the size of groups varies widely in nature and forms an implicit part of much experimental sexual selection research under laboratory conditions. Here I use simulations of mating competition within populations that vary in the size of groups they contain, to show that variation in group size, and in particular small groups, can influence sexual selection. Specifically, I show that null expectations for the operation of pre‐ and postcopulatory sexual selection is governed by the size of groups within populations because smaller group sizes constrain the structure of sexual networks leading to reinforcing episodes of pre‐ and postcopulatory sexual selection. Given broad variation in group structure in nature and the tendency for experimental sexual selection research to study replicate small groups, these effects have implications for our understanding of the operation of sexual selection in polyandrous populations.

## Graphical abstract

A key goal in sexual selection research is to understand the role that polyandry plays in shaping the strength of sexual selection and the relationship between pre‐ and postcopulatory sexual selection. Here, I use simulations of mating competition within populations that vary in the size of groups they contain, to show that variation in group size, and in particular small groups, influences sexual selection. I show that the operation of pre‐ and postcopulatory sexual selection is governed by the size of groups within populations because smaller group sizes constrain the structure of sexual networks leading to reinforcing episodes of pre‐ and postcopulatory sexual selection. Given the tendency for experimental sexual selection research to study replicate small groups, these effects have implications for our understanding of the operation of sexual selection in polyandrous populations.

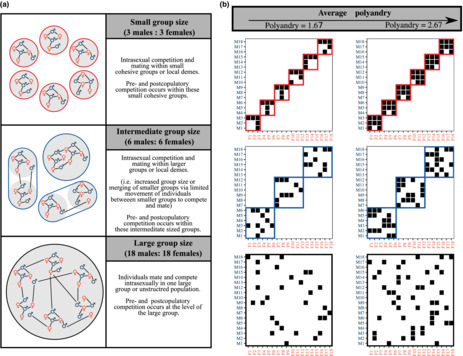

## 1 INTRODUCTION

Sexual selection arises from competition between members of the same sex to fertilize the gametes of the opposite sex and is responsible for a vast array of ornaments, weapons, and behaviors (Andersson, 1994; Darwin, 1871; McCullough et al., 2016). Over the last 50 years our understanding of sexual selection, and how it varies between populations, has been advanced by two key developments. First, is the realization that when females copulate with multiple males over the time fertilization occurs (i.e., are polyandrous), sexual selection on males continues after mating, forcing males to compete not only over the number and fecundity of mates but also over the proportion of their mate's ova that they fertilize (Eberhard, 2009; Parker, 1970; Parker & Birkhead, 2013; Pizzari & Wedell, 2013; Simmons & Wedell, 2020). Second, is the growing understanding that patterns of sexual selection are strongly dependent on variation in the social environment (Clutton‐Brock, 2017; Emlen & Oring, 1977; Lyon & Montgomerie, 2012; Maldonado‐Chaparro et al., 2018; McDonald et al., 2013; West‐Eberhard, 1983).

Polyandry principally impacts the operation of sexual selection on males by firstly generating sexual selection on postcopulatory traits [e.g., sperm, ejaculate, and behavioral traits that increase male paternity share (Birkhead & Møller, 1998; Eberhard, 2009)], and secondly by influencing the strength of precopulatory selection by influencing variation in male mating success (Collet et al., 2012; Kvarnemo & Simmons, 2013; Morimoto et al., 2019; Parker & Birkhead, 2013). Understanding the forces that modulate the relative strength of pre‐ and postcopulatory episodes of sexual selection and the conditions that determine whether these episodes of selection reinforce or oppose each other, remains an ongoing challenge (Collet et al., 2012; Cramer, 2021; Devigili et al., 2015; Evans & Garcia‐Gonzalez, 2016; Lüpold et al., 2014; Marie‐Orleach et al., 2016; McDonald, Spurgin, et al., 2017; Morimoto et al., 2019; Pélissié et al., 2014; Turnell & Shaw, 2015).

The impact of the social environment on sexual selection has long been recognized, including variation in operational sex ratios and population density (Emlen & Oring, 1977; Janicke & Morrow, 2018; Kokko & Rankin, 2006). More recently, research has further demonstrated that animal populations are structured by complex social and sexual networks (Albery et al., 2021; Beck et al., 2021; Krause et al., 2014; Maldonado‐Chaparro et al., 2018; McDonald et al., 2019, 2020; Muniz et al., 2015; Oh & Badyaev, 2010; Ryder et al., 2008; Silk & Hodgson, 2021; Smith et al., 2023). The structure of such socio‐sexual networks (i.e., the patterning of sexual interactions among individuals) can influence sexual selection by shaping the relationship between male precopulatory mating success and the intensity of postcopulatory competition he faces (Fisher et al., 2016; Greenway et al., 2021; McDonald et al., 2013; McDonald & Pizzari, 2018; Muniz et al., 2015; Sih et al., 2009; Wey & Kelly, 2019). For example, if more polygynous males mate with on average the least polyandrous females (negative mating assortment) this can create a positive covariance between male mating success and male paternity share, because males successful in precopulatory competition may also face the lowest intensity of postcopulatory competition (McDonald & Pizzari, 2016; Sih et al., 2009). Such patterns are expected to accentuate the benefits of increased mating success (i.e., Bateman gradients) and accentuate sexual selection (Greenway et al., 2021; McDonald & Pizzari, 2018). Alternatively, if the males with the highest mating success copulate with the most polyandrous females (positive mating assortment), they may suffer the most intense postcopulatory competition. These patterns may reduce the overall variance in male reproductive success and indicate trade‐offs between pre‐ and postcopulatory competitiveness (Fisher et al., 2016; McDonald & Pizzari, 2018), promoting the emergence of alternative male reproductive tactics (Kvarnemo & Simmons, 2013). Patterns of mating assortment in nature and in captive populations have the potential to vary widely (Fisher et al., 2016; Greenway et al., 2021; McDonald & Pizzari, 2018; Morimoto et al., 2019; Wey & Kelly, 2019) and preliminary investigations suggest these patterns of mating assortment may be related to both the size of mating groups and levels of polyandry (McDonald & Pizzari, 2018). Assessing under what scenarios we may expect sexual networks to show positive, negative or no assortment is therefore an important step in understanding the conditions that determine whether episodes of sexual selection reinforce or oppose each other (Evans & Garcia‐Gonzalez, 2016; Greenway et al., 2021; McDonald, Spurgin, et al., 2017; McDonald & Pizzari, 2018; Morimoto et al., 2019).

Group structure is a widespread axis of variation in the social organization of populations, where individuals form tight social units or are subdivided into small local demes (hereafter “groups”; Farine et al., 2015; Krause & Ruxton, 2002; Rousset, 2004). Group structure in polyandrous populations can vary widely, in some species individuals compete sexually as part of large aggregations whereas in other species, including many highly social animals, individuals live, and compete sexually within small distinct cohesive groups (Clutton‐Brock, 2016; Grueter et al., 2020; Hanlon, 1998; Krause & Ruxton, 2002; Shuster & Wade, 2003; Sullivan, 1981; Thornhill, 1980). For instance, many polyandrous primates live in small cohesive groups with 2–8 adult males (Bradley et al., 2005; Dixson, 2018; Kowalewski & Garber, 2010). Similarly, some galliformes form small polygynandrous social units of between 2 and 28 individuals (Collias & Collias, 1967, 1996; Collias & Saichuae, 1967). In invertebrates, intrasexual competition and mating may occur within restricted small subunits with only a handful or few dozen of individuals, such as within the local demes of forked fungus beetles (*Bolitotherus cornutus*) and other insects (Formica et al., 2011; Greeff & Ferguson, 1999), as well as small local aggregations of hermaphroditic barnacles and leeches (Tan et al., 2004; Wilkialis & Davies, 1980). Such group structure is also an implicit component of much of sexual selection research under laboratory conditions, where small groups are experimentally constructed and where mating and competition is restricted to within these small groups (De Lisle & Svensson, 2017). For example, experimental studies of the impacts of polyandry in fowl (*Gallus gallus*) have used groups ranging from 3 males and 4 females to 10 males and 12 females (Collet et al., 2012; McDonald, Spurgin, et al., 2017; Roth et al., 2021). In mammals, experimental investigations of sexual selection have used group sizes of 2 females and 2–4 males (Mills et al., 2007), while studies in fish and reptiles have investigated the effects polyandry, sex ratios, and density on sexual selection in groups ranging from 2 to 60 individuals (Aronsen et al., 2013; Devigili et al., 2015; Fitze & Le Galliard, 2008; Head et al., 2008; Wacker et al., 2013). In invertebrates, the group sizes employed also vary between model species, including 5 individuals in hermaphroditic snails and flatworms (Hoffer et al., 2017; Marie‐Orleach et al., 2016; Pélissié et al., 2014), from 3 males and 3 females to 16 males and 16 females in *Drosophila* species (Bateman, 1948; Bjork & Pitnick, 2006; Gowaty et al., 2012; Morimoto et al., 2019; Pischedda & Rice, 2012), 10 males and 10 females in Squash bugs (Greenway et al., 2021) and 20 males and 20 females in swordtail crickets (*Laupala cerasina*; Turnell & Shaw, 2015). As a result, many sexual selection studies implicitly emulate populations structured into groups, whether assessing patterns of sexual selection within groups or overall patterns of selection across groups (De Lisle & Svensson, 2017). While the consequences of constraining interactions in animal social networks to within small groups has been considered in terms of patterns of infectious disease transfer (Nunn et al., 2015; Sah et al., 2018) as well as phenotypic assortment and social selection (McDonald, Farine, et al., 2017), the effects of group structure on the scope for different patterns of sperm competition in sexual networks and its impact on pre‐ and postcopulatory sexual selection remains comparatively unexplored (Rodriguez‐Exposito & Garcia‐Gonzalez, 2021).

**FIGURE 1:**
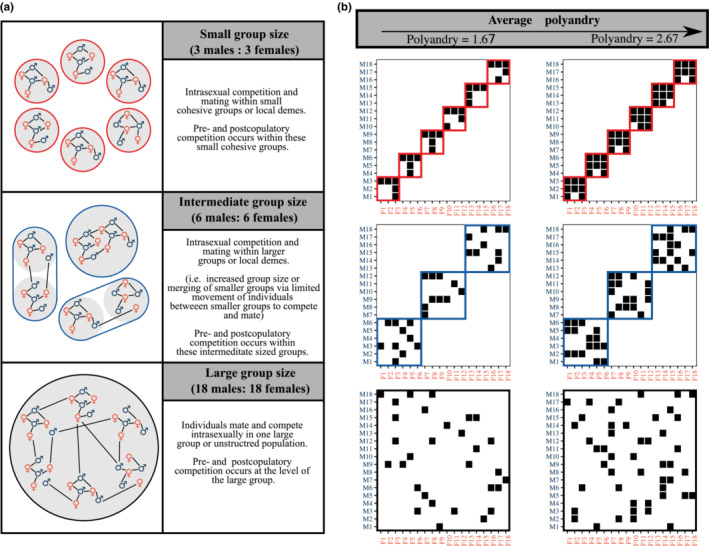
Variation in competitive group size in polyandrous populations. (a) Diagrams show three idealized polyandrous populations of males and females. The sexual networks of each population are shown where males (blue ♂) and females (orange ♀) are nodes connected by links representing copulations. Colored lines encircle competitive groups where individuals mate and compete intrasexually for fertilizations and therefore the scale at which pre‐ and postcopulatory competition occur. Populations range from small groups; a where intrasexual competition and mating occur within groups of three males and three females (red circles), medium groups; an intermediate case with larger groups of six males and six females (blue circles), to one large group of 18 males and 18 females (black circle) where all males and females freely interact, and competition occurs at the level of the population or otherwise large aggregation. (b) Matrix representations of the sexual networks of hypothetical populations of 18 males (rows) and 18 females (columns) with three different levels of group structure. Colored rectangles encircle competitive groups including (red) six small groups, (blue) three medium sized groups or (black) one large group. Black squares indicate pairs that copulated. From left to right each population represents one of two levels of average polyandry (i.e., the mean number of mating partners per female) including either 1.67 or 2.67 male mating partners per female on average.

Here I address this using simulated populations to investigate how the size of groups within group‐structured polyandrous populations can impact on the relationship between pre‐ and postcopulatory sexual selection on males. I used using simulated populations of equal size and sex ratio but which vary in the size of sexually competitive groups. Specifically, I generate replicate populations where males compete for fertilizations within small groups (3 males:3 females), intermediate groups (6 males:6 females) or one large group (18 males:18 females) at different levels of average female polyandry (i.e., average number of mating partners per female; Figure 1). I show that group structure constrains the structure of the sexual network, such that when mating competition occurs within smaller groups, this leads to (i) negative patterns of mating assortment and reinforcing episodes of pre‐ and postcopulatory sexual selection and (ii) a stronger potential for sexual selection on male precopulatory traits. These results provide testable null expectations for the effect of group size on pre‐ and postcopulatory sexual selection. Given the tendency for experimental sexual selection research to study small replicate groups, these effects may have implications for our understanding of the operation of sexual selection in polyandrous populations.

## 2 MATERIALS AND METHODS

To explore the impact of different group sizes on sexual networks and the relationship between pre‐ and postcopulatory sexual selection, I first constructed artificial populations of 18 males and 18 females. In each population, individuals mated within small groups of three males and three females with one of five predefined mating distributions that differed in average polyandry (i.e., the mean number of male mating partners per female) ranging from 1.67 to 2.67 mates per female, and also differed in the standardized variance in male mating success (i.e., the opportunity for precopulatory sexual selection, $I_{M}={\mathrm{VAR}}_{M}/{\bar{M}}^{2}$; Appendix 1). The range of average polyandry utilized here is biologically relevant given both behavioral and extra‐pair paternity studies indicate that the average number of mates per female often ranges between 1 and 2 males in primates (Qi et al., 2020; Reichard, 1995), in birds (Brekke et al., 2013; Dunn et al., 2009; Fiske & Kålås, 1995; Grinkov et al., 2022; Krietsch et al., 2022; Webster et al., 1995; Wetton et al., 1997), as well as in studies of polyandrous spiders and reptiles (Levine et al., 2015; Watson, 1998). Moreover, the range of polyandry per female of 1–3 mates in simulations represents the minimum and maximum possible levels of female polyandry when group sizes are constrained to within small groups of 3 males and 3 females, similar to experimental studies in systems such as flies, voles, and fowl (Collet et al., 2012; Mills et al., 2007; Morimoto et al., 2019). I generated 100 random starting populations for each of the five predefined mating distributions (Appendix 1). These initial starting populations therefore provide example populations (i) where individual males mate and compete within small groups of six individuals, comparable to the lower end of the freely mating group sizes often used in experimental studies of sexual selection, (ii) with different levels of polyandry and (iii) with a range of levels of male mating skew.

For each population, I constructed its sexual network where individual males and females are nodes connected by edges (links) if they copulated (Figure 1). To investigate patters of network mating assortment, I first calculated each male's sperm competition intensity (SCI). A male's SCI can be described by the average mating success of his female partners in the network of male–female copulations (i.e., the average polyandry of his partners, [McDonald & Pizzari, 2016; Shuster & Wade, 2003]) calculated as ${\mathrm{SCI}}_{i}=1/\frac{1}{M_{i}}\left(\sum_{j}^{M}1/k_{j}\right)$, where *M*_*i*_ is the number of females the *i*_th_ male mated with (i.e., mating success) and *k*_*j*_ is the mating success of his *j*_*th*_ female mating partner (McDonald & Pizzari, 2016; Shuster & Wade, 2003). SCI thus represents the harmonic mean mating success of a male's female partners (e.g., where a value of one means a male has complete exclusivity with his female partners, a value of two means his female partners on average mate with two males and so he sperm competes with on average one rival male).

I then measured each population's network mating assortment as the relationship between a male's mating success (number of unique mating partners) and his SCI, termed the sperm competition intensity correlation (SCIC; McDonald & Pizzari, 2016, 2018). In a population where the polyandry of a male's female partners impacts on his paternity share (e.g., higher SCI reduces a males paternity share by increasing sperm competition), SCIC is expected to impact patterns of sexual selection. For example, consider a population with positive selection on male mating success, that is, a positive male Bateman gradient ($\beta_{M}$), where $\beta_{M}$is calculated as $T=\beta_{M}M+\varepsilon$, and ε is an error term. A male's SCI can be added to the above regression such that $T=\beta_{M∙\mathrm{SCI}}M+\beta_{\mathrm{SCI}∙M}\mathrm{SCI}+\varepsilon$. Here $\beta_{M∙\mathrm{SCI}}$ represents a partial sexual selection gradient on male mating success controlling for variation in SCI and $\beta_{\mathrm{SCI}∙M}$ represents a partial sexual selection gradient on male SCI controlling for variation in male M. In general, sexual selection theory predicts that increasing sperm competition will decrease a male's reproductive success (i.e., a negative $\beta_{\mathrm{SCI}∙M}$), whereas male mating success increases his reproductive success (i.e., a positive $\beta_{M∙\mathrm{SCI}}$). As a result, the way in which the Bateman gradient, $\beta_{M}$, is affected by variation in sperm competition intensity across males is determined by the slope of the regression of each male's sperm competition intensity on male mating success (*M*; i.e., the sperm competition intensity correlation, SCIC) such that $\beta_{M}=\beta_{M∙\mathrm{SCI}}+(\text{SCIC}\times \beta_{\mathrm{SCI}∙M}$; McDonald & Pizzari, 2016). Strong positive values of SCIC may mean males with high mating success face high sperm competition and generate a negative covariance between mating success and paternity share (negative COV_MP_) and therefore counteracting pre‐ and postcopulatory episodes of sexual selection and a reduced $\beta_{M}$ (Fisher et al., 2016; McDonald & Pizzari, 2016, 2018; Sih et al., 2009). Whereas strong negative values of SCIC may generate a positive covariance between mating success and paternity share (positive COV_MP_), and therefore reinforcing pre‐ and postcopulatory episodes of sexual selection and stronger positive values of $\beta_{M}$ (Greenway et al., 2021; McDonald & Pizzari, 2016, 2018).

For each starting population I then conducted randomizations to vary the structure of the sexual network, to either increase or decrease the SCIC. Crucially, to explore the effects of group structure, I conducted the above stepwise randomizations in three different ways that allowed mating to occur within increasingly larger group sizes. This included mating within (i) small groups; where all randomized swaps occur only within the original predefined groups of three males and three females, (ii) medium groups; where all randomized swaps occur within groups of six males and six females obtained by merging pairs of small groups, and (iii) large groups; where randomized swaps were made between pairs across the whole population (i.e., one large group or unstructured population of 18 males and 18 females). All randomizations hold constant the mean polyandry and the variation in male and female mating success of the original population. Randomizations were conducted in a stepwise way for each population, one pairwise swap at a time, and retained each newly assorted population and continued until randomizations could no longer increase or decrease the SCIC. This process means that for each mating distribution (i.e., level of polyandry and $I_{M}$) we now have populations of equal size where males and females mate and compete within group sizes ranging six individuals (3 males:3 females), 12 individuals (6 males:6 females), or 36 individuals (18 males:18 females), comparable to the range of the freely mating group sizes used in experimental studies of sexual selection, and each group size also has range of levels of SCIC. Such a simulation approach allows exploration of different network structures given differences in group size, as well differences in average and variance in mating rates within the network and is commonly applied in studies of assortative patterns of connections in networks (Farine, 2014; Montiglio et al., 2018). In total, this process generated 11,843 randomized mating populations across all five mating distributions.

For every population I then simulated sperm competition, where each female produced 10 ova and every male that mated with a female had an equal probability of fertilizing each of her ova (i.e., if two males mated with a female, each male had a 50% chance of fertilizing each ova). Under this scenario sperm competition occurs under a fair raffle where each male has an equal sperm contribution, and therefore represents a simple null model that does not rely on assumptions of male competitive phenotypes (Parker & Pizzari, 2010). For every population, I then calculated each male's paternity share (P) as the proportion of all his partners' ova that he fertilized and the covariance between a male's mating success and his paternity share (COV_MP_) following Webster et al. (1995). Holding all else constant, a higher positive value of COV_MP_ will result in a higher positive covariance between mating success and reproductive success (i.e., COV_MT_), and therefore a steeper positive Bateman gradient ($\beta_{M}$). I therefore also calculated the mean standardized male $\beta_{M}$ for each population, where a mean standardized Bateman gradient of 1 indicates that a 100% increase in relative mating success results in a 100% increase in relative reproductive success and allows direct comparisons between populations (Arnqvist, 2013; Hereford et al., 2004). In addition, I calculated the maximum potential sexual selection gradient on a male precopulatory trait (i.e., the Jones Index = $\beta_{M}\sqrt{I_{M}}$; Jones, 2009). The Jones index combines information on both the variation in male mating success (*I*_*M*_) and the mean standardized Bateman gradient and has been more recently indicated as an accurate measure of the strength of sexual selection on phenotypic traits (Henshaw et al., 2016). Holding $I_{M}$ constant, a steeper positive Bateman gradient will result in larger values of the Jones index and a higher potential for sexual selection on a male precopulatory trait (Henshaw et al., 2016; Jones, 2009). Similarly, higher values of $I_{M}$ will result in a higher Jones index, as there is a stronger potential for sexual selection to act on variation in male mating success.

Finally, for each of these three related metrics SCIC, COV_MP_ and the Jones Index, I constructed linear mixed‐effects models to compare how they varied as a function of both group size and polyandry. Each model included group size as a 3‐level factor, average polyandry as a continuous variable, their interaction and the identity of original starting population as a random effect. The significance of interactions was assessed by comparing models with and without interactions using likelihood ratio tests. All analysis was conducting using R statistical software version 4.2.1 (R Core Team, 2022) and mixed‐effects models using package “lme4” (Bates et al., 2015).

## 3 RESULTS

**FIGURE 2:**
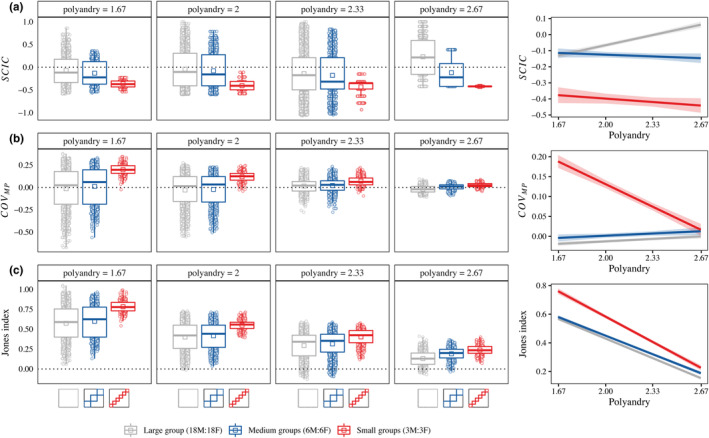
Group size impacts the relationship between pre‐ and postcopulatory episodes of sexual selection. Boxplots show (a) the sperm competition intensity correlation (SCIC), (b) the covariance between male mating success and male paternity share (COV_MP_) and (c) the maximum potential sexual selection gradient on male precopulatory traits (Jones Index) calculated for simulated for populations of 18 males and 18 females (*n* = 11,843) with four different levels of average polyandry (i.e., mean number of mating partners per female = 1.67, 2, 2.33, or 2.67) and three levels of group structure, small groups (3 males:3 females, red), equally sized medium groups (6 males:6 females, blue) or one large group or unstructured population (18 males:18 females, gray). Points behind boxplots show individuals values for each simulated population and squares show mean values. Line graphs show predictions from linear mixed‐effects models for the effect of polyandry and group size on each metric. Shaded areas represent 95% confidence intervals.

Across all levels of polyandry, SCIC was significantly lower and was restricted to negative values when males competed in small groups, compared to medium and larger groups, where variation in SCIC was larger and spanned both positive and negative values (Figure 2a, Table 1). There was a significant interaction between group size and polyandry ($\chi_{2}^{2}$ = 87.174, *p* < .001, Table 1) driven by the absence of extremely negative SCICs in large groups at highest level of polyandry. As expected, SCIC was negatively associated with COV_MP_ because in simulated populations with a positive SCIC, males with the highest mating success (M) must share their paternity (P) with more rivals, reducing the covariance between M and P, and consequently reducing male Bateman gradients (Appendix 2).

**TABLE 1:** Linear mixed‐effects model results for the effects average polyandry and group size on the sperm competition intensity correlation (SCIC), the standardized covariance between mating success and paternity share (COV_MP_) and the maximum potential precopulatory selection gradient (Jones index).

Parameter	Response		
	SCIC	COV_MP_	Jones index
[Intercept] Group size (Large)	−0.448 [−0.518 to −0.378]	−0.050 [−0.071 to −0.029]	1.257 [1.222 to 1.292]
Group size (Intermediate)	0.389 [0.271 to 0.507]	0.018 [−0.021 to 0.057]	−0.023 [−0.066 to 0.021]
Group size (Small)	0.179 [−0.002 to 0.360]	0.524 [0.464 to 0.584]	0.394 [0.328 to 0.461]
Polyandry × Group size (Large)	0.191 [0.159 to 0.223]	0.019 [0.009 to 0.028]	−0.414 [−0.430 to −0.398]
Polyandry × Group size (Intermediate)	−0.224 [−0.278 to 0.169]	−0.002 [−0.020 to 0.016]	0.021 [0.001 to 0.042]
Polyandry × Group size (Small)	−0.256 [−0.337 to −0.174]	−0.190 [−0.217 to −0.163]	−0.121 [−0.151 to −0.091]

*Note*: Effects are shown compared to large group size as the reference level, alongside upper and lower 95% confidence intervals. All interactions are significant at the *p* < .001 level.

As a consequence of the bias toward negative SCIC values in small groups, COV_MP_ was significantly higher and largely restricted to positive values in small groups for all levels of polyandry, whereas in medium and large groups, COV_MP_ was typically lower and spanned both positive and negative values (Figure 2b). The absolute magnitude of COV_MP_ was constrained toward zero at higher levels of polyandry as a consequence of reduced variation in male mating success and paternity share (Figure 2b). In addition, there was a significant interaction between polyandry and group size ($\chi_{2}^{2}$ = 193.620, *p* < .001, Table 1) as the range of COV_MP_ decreased with polyandry toward zero in small groups, while the contraction towards zero in large groups resulted in an overall increase in average COV_MP_ (Figure 2b).

Finally, for all levels of polyandry, group size significantly impacted on the Jones index, with smaller groups consistently displaying a higher Jones index due to higher values of male COV_MP_ and $\beta_{M}$ compared to medium or large groups (Figure 2c). The Jones index decreased with increasing levels of polyandry and its associated lower variance in mating success, but this rate of decrease was faster in small groups ($\chi_{2}^{2}$ = 74.776, *p* < .001, Figure 2c, Table 1).

## 4 DISCUSSION

Many animals live and compete for fertilizations in populations subdivided into small groups or local demes (Collias & Collias, 1996; Farine et al., 2015; Formica et al., 2011; Krause & Ruxton, 2002; Rousset, 2004; Shuster & Wade, 2003; Tan et al., 2004; Wilkialis & Davies, 1980) and much of sexual selection research under laboratory conditions, examines sexual selection in small experimentally constructed groups (De Lisle & Svensson, 2017). In this study, I used a simulation approach to investigate how variation in the size of such groups may impact on patterns of sexual selection through its effect on the structure of sexual networks.

The results presented here firstly highlight that group size can impact on patterns of mating assortment in sexual networks, by constraining the range of SCIC (i.e., smaller groups more strongly limit the range of possible network structures). Specifically, by limiting mating to among only a few individuals, smaller groups restrict SCIC to more negative values (i.e., where the more successful males also copulate with the least polyandrous females, whereas the least successful males typically mate with the most polyandrous females), even for populations with the same mean and variance in mating rates. This effect is similar to limitations in the diversity of social interactions in social networks (Kappeler, 2019). In social networks, the number of individuals that a focal individual can interact with increases linearly as additional individuals are added to the network. However, in contrast the number of potential interacting pairs increases exponentially (Kappeler, 2019). In terms of sexual interactions in sexual networks, doubling group size from two males and two females to four males and four females, adds two new possible mates and competitors for each male individual, but increases the number of possible mating pairs from 4 to 16. As a result, this means that the number possible mating pairs of males and females (i.e., the mating matrix) is far fewer in populations structured into small groups versus even moderately larger groups (i.e., Figure 1b), restricting possible patterns of assortment.

As a consequence of limiting patterns of mating assortment toward more strongly negative values, populations with small groups consistently demonstrated a higher, more positive COV_MP_ compared to populations with larger groups, across levels of polyandry. This is because in negatively assorted populations, males with the highest mating success on average also have the highest exclusivity with their mates, whereas males with the lowest mating success copulate on average with the most polyandrous females. The simulations here assume a simple raffle‐type sperm competition as a null model (Parker & Pizzari, 2010), where each male has equal sperm contribution and therefore increases in a male's sperm competition (SCI) as a result of female polyandry in will result in a reduction in male paternity share (Greenway et al., 2021; McDonald & Pizzari, 2018). Given there are no male traits or cryptic female choice within simulations, systematic variation in COV_MP_ can only emerge via the structure of mating networks, that is, SCIC. A key advance of the results presented here is therefore to demonstrate that for populations with the same mating distributions (i.e., mean and variation in male and female mating success), null expectations for patterns of SCIC and COV_MP_ are impacted by group size alone. The impact of SCIC on COV_MP_ then has downstream consequences both for Bateman gradients and therefore ultimately the maximum potential selection on precopulatory traits (i.e., the Jones index). These results therefore provide null expectations for the relationship between pre‐ and postcopulatory episodes of sexual selection and show that null expectations vary based on group size alone. Appreciating such null expectations is likely crucial to avoid misinterpretations when extrapolating patterns observed in small groups to larger groups and when comparing between study systems and experiments using different group sizes, particularly as positive values COV_MP_ of may arise purely due to small group sizes.

In nature, a diversity of sperm competitive mechanisms may exist (Parker & Pizzari, 2010) and future studies should further test the impact of such different mechanisms on null patterns of sexual selection. Despite this, consistent with the results presented here, experimental studies that have estimated COV_MP_ in small replicate polyandrous groups, have typically identified either positive or weakly positive values of COV_MP_ (Collet et al., 2012; Devigili et al., 2015; Marie‐Orleach et al., 2016; McDonald, Farine, et al., 2017; Pélissié et al., 2014; Turnell & Shaw, 2015), although some studies have produced more variable positive and negative values of COV_MP_ (Morimoto et al., 2016, 2019). While the simulations presented here included only subset of the full possible range of mating distributions, these results indicate that for populations with a similar *I*_*M*_ and average polyandry, males with low mating success in populations structured into small groups may typically face stronger sperm competition intensities and these populations will be characterized by pre‐ and postcopulatory episodes that act in concert (i.e., a positive COV_MP_). Such effects may accentuate the reinforcement of traits such as male social dominance that have been shown to be favored by both pre‐ and postcopulatory sexual selection in several species such as cockroaches and in small groups of fowl (Montrose et al., 2008; Pizzari & McDonald, 2019). Future research should further investigate how the traits targeted, and relative strength of pre‐ and postcopulatory sexual selection on such traits, differs among populations subdivided into groups of different sizes.

As a result of the typically stronger reinforcement between pre‐ and postcopulatory episodes in small groups, the simulations presented here further show that small groups had consistently higher maximum potential strength of sexual selection on precopulatory traits (Jones index) across all levels of polyandry. Moreover, these results also show that as polyandry increases, the range of both the Jones index and COV_MP_ consistently reduces. This highlights how in smaller groups, increases in polyandry may rapidly saturate the number of possible mating pairs in a population structured into smalls groups, more dramatically eroding variation in male mating success and the magnitude of such covariances. For example, in groups where average female polyandry is three male mates per female, this may represent strong sperm competition levels regardless of whether the population is unstructured or is divided into local mating groups of three males and three females. However, in the population structured into groups of three males and three females, there would be no variation in male mating success, and variation in male reproductive success would instead be driven only by postcopulatory mechanisms. Crucially, such group sizes and levels of polyandry are not unusual in both nature and experimental settings (e.g., Bjork & Pitnick, 2006; Collet et al., 2012; Collias & Collias, 1967; Collias & Saichuae, 1967; House et al., 2019; Krause & Ruxton, 2002; Mills et al., 2007; Morimoto et al., 2019; Oklander et al., 2014). For example, a recent experimental investigation of Bateman gradients in the hermaphroditic pond snail (*Lymnaea stagnalis*) explored polyandrous groups of five individuals, in which variation in mating success was quickly eroded as every individual had soon copulated with every partner in both male and female roles in the early stages of the experiment (Hoffer et al., 2017). Similarly, in an explicit examination of the effects of group size on mating system plasticity in water striders (*Aquarius remegis*), individual groups of a population subdivided into units of three males and three females in some cases reached saturation of the mating matrix with up to 88%–100% of possible mating pairs copulating in only 6 days (Sih et al., 2017). Taken together the results presented here suggest that understanding the role of precopulatory sexual selection across experiments requires an appreciation of how polyandry differentially affects variation in mating success in populations characterized by different group sizes. In other words, to understand how polyandry impacts sexual selection it is crucial to appreciate that an increase in average polyandry of one or two male partners per female in a population structured into small groups may result in a qualitatively different impact on the potential for precopulatory sexual selection compared to large aggregations or more openly mixed populations. Future experimental research should explicitly address how variation in polyandry impacts on sexual selection across populations with differing group structures and group sizes.

More broadly these results also demonstrate that null expectations for the relationship between pre‐ and postcopulatory sexual selection (COV_MP_) can be non‐zero and secondly that these null expectations vary depending on the group structure of populations (i.e., more positive COV_MP_ in populations structured into small groups). This suggests that future research investigating the relationship between pre‐ and postcopulatory episodes of sexual selection and their contribution to sexual selection, should generate null expectations that may be non‐zero and assess observed patterns in the context of these null expectations. Ideally, careful experiment should complement the use of null models. For instance, while mating patterns may not differ in comparison with random networks, experiment may more effectively assess the extent to which observed patterns are a result of random processes versus counteracting non‐random behavioral mechanics. Exploring sexual networks in systems that allow manipulations that exclude individual competitive mechanisms, such as those that prevent males from delivering mating plugs, or block the impact of male seminal fluid proteins, may be particularly useful (e.g., Morimoto et al., 2019).

The results here indicate that the strongest impact of group structure should be observed when sexual competition is consistently limited to within small groups either via experiment or in natural populations. In nature a wide variety of factors can place limits on the size of groups within populations. For example, foraging strategy, prey density, predation pressure, ecological constraints, social competition, and risk of infectious disease spread, may all restrict the minimum and maximum size of groups (Chapman & Chapman, 2000; Creel et al., 2014; Hamilton, 1971; Janson, 1988; Kasozi & Montgomery, 2020; Lucchesi et al., 2020; Markham et al., 2015; Nunn et al., 2015; Szemán et al., 2021; Takada & Washida, 2020; Teichroeb & Sicotte, 2009). However, in many natural populations group membership may be more temporally fluid. For example, when extra‐group matings are common and/or multiple groups are more highly interconnected (e.g., Cant et al., 2002; Carpenter et al., 2005; Lucchesi et al., 2020) or when females visit and copulate at multiple leks rather than mating within one lek (Hess et al., 2012; Schroeder, 1991). This movement and mating between groups may effectively increase the size of the group in which competition occurs. When competition and mating is sufficiently fluid between social groups, the scale at which competition occurs will instead more closely match that of non‐group structured more openly mixed population. As a result, if group sizes in nature are typically larger than those used in experiments, the results here suggest scope for a potential disconnect between patterns of pre‐ and postcopulatory sexual selection observed in laboratory experiments and patterns expected in larger natural groups.

Finally, while I have discussed the impact of increasing polyandry on patterns of sexual selection on males in populations that vary in group structure, group structure may itself impact on levels of polyandry. For example, the division of populations into smaller groups may favor an increase in female polyandry to avoid the negative consequences of local group male infertility (Dean et al., 2010). Alternatively, if males that copulate frequently are more sperm‐limited (Warner et al., 1995; Wedell et al., 2002)—all else being equal—successful males structured within small groups may be comparatively less sperm‐limited than successful males in unstructured populations where access to mates is less restricted. As a result, small group sizes may reduce female sperm limitation and disfavor increases in polyandry. Understanding the frequency of such alternate scenarios my provide new insights into the potential coevolution of group size and polyandry.

## 5 CONCLUSION

A key goal in sexual selection research is to understand the role that polyandry plays in shaping the strength of sexual selection (Birkhead & Pizzari, 2002; Eberhard, 2009; Parker & Birkhead, 2013; Pizzari & Wedell, 2013; Simmons & Wedell, 2020) and the relationship between pre‐ an postcopulatory sexual selection (Evans & Garcia‐Gonzalez, 2016; Kvarnemo & Simmons, 2013). Here I have shown how group size may influence the structure of sexual networks with downstream consequences for the relationship between pre‐ and postcopulatory episodes of sexual selection. These insights indicate that the strongest impact of polyandry should be observed when mating is consistently limited to within small groups, such as within in experimentally defined small mating groups or in nature where species form tight polygynandrous social units. The predictions outlined here are readily testable in future studies by using manipulations of group size in experimental settings. In addition, such studies should explore to what extent such effects may be altered by variation in groups size within populations. Finally, I suggest that future studies exploring the impact of polyandry on sexual selection should explicitly consider variation in group sizes when comparing between systems. It seems likely that experimental studies focusing on small replicate groups represent the extreme end of the impact of polyandry.

## AUTHOR CONTRIBUTIONS

**Grant McDonald:** Conceptualization (lead); formal analysis (lead); funding acquisition (lead); investigation (lead); methodology (lead); visualization (lead); writing – original draft (lead); writing – review and editing (lead).

## CONFLICT OF INTEREST STATEMENT

The author declares no competing interests.

## Data Availability

Data are available at https://doi.org/10.6084/m9.figshare.22663924.v1.

## APPENDIX 1

### 1.1

Five example starting mating matrices representing each of the five unique male mating distributions used for simulations described in the main text (a–e). The mating matrix for each population has 18 males and 18 females, where rows represent individual males (M1–M18) and columns represent individual females (F1–F18). Black squares indicate that a pair copulated. The average polyandry (mean number of male mating partners per female) and opportunity for precopulatory sexual selection on males (*I*_*M*_) for each mating distribution is given. Colored squares outline the original small groups of three males and three females in which mating occur, prior to randomizations used to generate different levels sperm competition intensity correlations (SCIC) and different group sizes. Stepwise randomizations were conducted in three ways that allowed mating to occur within groups of different sizes; included limiting randomized swaps to within (i) the original predefined groups of three males and three females (small groups), (ii) within groups of six males and six females obtained by merging pairs of small groups (medium groups), and (iii) one large group of 18 males and 18 females, where randomized swaps were made between pairs across the whole population. Stacked histograms show the frequency distributions of male mating success (M) for each population, and colors indicate the frequency distributions of male mating success within each original small group in each population. Figure shows *I*_*M*_ calculated using the sample variance.

## APPENDIX 2

### 2.1

Relationship between the sperm competition intensity correlation (SCIC) and (a) the covariance between male mating success and male paternity share (COV_MP_) and (b) mean standardized male Bateman gradients ($\beta_{M}$) for simulations described in the main text. Each point represents a value calculated for an individual population of 18 males and 18 females (*n* = 11,843). Results for populations at different levels of average polyandry (mean number of mated per female = 1.67, 2.00, 2.33 and 2.67) are shown on each panel. Colors indicate the group structure of the population, that is, where mating was restricted to occur within either groups of three males and three females (small groups, red), groups of six males and six females (medium groups, blue), and one large group of 18 males and 18 females (large groups, gray). Higher and more positive levels of SCIC predict lower and more negative COV_MP_, however higher levels of polyandry flatten the relationship between SCIC on COV_MP_ (a). Higher and more positive levels of SCIC predict lower $\beta_{M}$, however at higher levels of polyandry the predictive power of SCIC on $\beta_{M}$ is reduced (b). For all levels of polyandry, the scope for variation in SCIC is limited by smaller groups versus larger groups, resulting in more positive COV_MP_ and a steeper $\beta_{M}$ in small groups.
